# Inhibitors of Proteases: A Well-Grounded Strategy in Drug Development

**DOI:** 10.3390/molecules30142909

**Published:** 2025-07-10

**Authors:** Santo Previti, Roberta Ettari

**Affiliations:** Department of Chemical, Biological, Pharmaceutical, and Environmental Sciences, University of Messina, Viale Stagno d’Alcontres 31, 98166 Messina, Italy; spreviti@unime.it

Enzymes are biocatalysts that are widespread in living organisms. Structurally, an enzyme is a protein that is able to bind with specific substrates, which are converted into different molecules called products [1]. The majority of metabolic processes in cells need enzymes, which are required to occur at optimal rates in order to sustain the life of the cell itself. Therefore, the suitable functionality of enzymes is essential to maintain the catalytic efficiency, substrate specificity, and regulatory control required for cellular viability and homeostasis [1].

In humans, enzyme dysregulations and malfunctions are often associated with a pathological state [2,3]. Similarly, overly catalytic activity in pathogens, such as bacteria, viruses, protozoa, and fungi, enhances the pathogenesis, infectivity, resistance, and survival of infective agents [4,5].

For all these reasons, the scientific community is intensively involved in the development of enzyme inhibitors to properly modulate their catalytic activity.

Among all the enzyme classes, the possibility of properly modulating enzymes that catalyze proteolysis, breaking down proteins into smaller peptides or single amino acids, was found to be of particular interest [6]. Proteases, also known as peptidase or proteinase, or proteolytic enzymes, cleave the peptide bonds by means of a nucleophilic agent attack on the peptidyl carbonyl group. Seven broad groups of proteases were listed based on the nucleophilic agent, namely cysteine, serine, threonine, aspartic, glutamic, asparagine, and metallo-proteases [7].

Generally, inhibitors of proteases bind to the enzyme catalytic site, resulting in the loss of proteolytic activity. Four different classes of inhibitors can be categorized based on their mechanism of action: competitive, non-competitive, irreversible, and transition-state analogs [8,9]. Structurally, the inhibitors of proteases are peptides and peptidomimetics, which show great similarity to the natural substrate or small molecules [10,11].

To date, 641 proteases with crucial roles in diverse biological processes, such as protein turnover, cell signaling, homeostasis, and immune response, among others, have been found in humans [12]. The World Health Organization (WHO) revealed the top ten causes of death in 2021, which included ischemic heart diseases, pulmonary diseases, cancer, diabetes, and kidney diseases [13]. Regarding heart and kidney diseases, angiotensin-converting enzyme (ACE) is a protease whose inhibition results in a reduction in blood pressure, with consequent benefit for patients [14,15]. For pulmonary diseases, such as chronic obstructive pulmonary disease (COPD), the higher expression and activity of serine proteases, like neutrophil elastase and proteinase, are associated with increased severity. Therefore, the inhibitors of these proteases lead to the benefits of pulmonary functions [16,17]. In relation to cancer, proteases are actively involved in tumorigenesis and tumor progression because aberrant proteolytic activity results in altered substrate cleavage [18]. The development of inhibitors of cathepsins, proteasome, and kallikreins, among others, could reduce tumor severity and prevent metastasis [19,20,21]. A panel of proteases like calpains, cathepsin, caspases, and dipeptidyl peptidase-4 (DPP-4) take an active part in hyperglycemia and the micro- and macrovascular complications related to diabetes. In particular, DPP-4 inhibitors, commonly known as gliptin, have been largely developed [22]. In contrast to the previously mentioned diseases, the role of inhibitors of different types of hydrolases, such as lipase [23], amylase [24], and phosphatase [25], has been thoroughly investigated in diabetes.

Regarding viruses, the SARS-CoV-2 pandemic has further highlighted that the identification of inhibitors targeting proteases is a successful strategy for the development of antiviral agents. In fact, two years after the first appearance of the virus, nirmatrelvir, a SARS-CoV-2 main protease inhibitor, was approved for the treatment of non-severe forms of COVID-19, in combination with ritonavir (Paxlovid^®^) [26]. Another relevant research field in which protease inhibition has yielded significant breakthroughs is the treatment of HIV. In particular, HIV-1 protease (PR) is considered an attractive target for the development of antiretroviral drugs [27]. From 1995 to the present, nine HIV-1 PR inhibitors have been marketed. Unfortunately, their long-term effectiveness and side effects limit HIV treatment. In light of this, substantial and ongoing efforts are being dedicated to the development of more potent and effective antiviral drugs against HIV [28,29,30].

With regard to protozoa, several diseases, such as malaria, amebiasis, giardiasis, toxoplasmosis, leishmaniasis, American trypanosomiasis, and sleeping sickness, pose a constant threat to billions of people, especially in the southern hemisphere [31]. Numerous studies have demonstrated that protozoal proteases are valid targets for the development of antiprotozoal agents. Starting from this, potent inhibitors of protozoal proteases like falcipains, *Lm*CPB2.8, giardipain-1, *Tg*CPL, GP63, cruzain, and rhodesain have been reported in the literature [32,33,34,35,36].

As for bacteria, the emergence of bacteria that are resistant to multiple classes of antibiotics is particularly troubling [37]. The golden age of antibiotics is gone, and the discovery of alternative targets and novel and effective drugs against bacteria will be one of the biggest challenges for the coming years [38]. Bacterial proteases are crucial enzymes and potential therapeutic targets for antimicrobial drug development. Proteases presenting in both Gram-negative and Gram-positive bacteria are of particular interest. Among them, ATP-dependent Clp, Lon, and FtsH proteases, HtrA, and HslVU have been largely studied, resulting in the identification of potent inhibitors [39,40,41].

Concerning fungal infection, the use of antifungal medication is often associated with significant side effects, long healing treatments, drug interactions, and resistance. Proteases have also been found to be valid targets for the development of novel antifungal agents in this field [42]. In particular, serine proteases have largely been characterized, and several peptide-based inhibitors with different reactive portions, commonly known as warheads, are present in the literature [42]. Naturally sourced compounds were found to be of particular interest, both for biological characterization and as a starting point for SAR studies and drug-like optimization [43].

As previously stated, side effects and drug resistance represent two significant drawbacks in therapy. In order to overcome these limitations, the use of drugs in combination, as well as the rational development of inhibitors aimed at two different targets, were found to be valid approaches. In fact, drugs in combination could lead to several advantages, such as increased treatment effectiveness, a reduced risk of drug resistance onset, synergistic effects, and the use of lower doses, and therefore, reduced side effects [44,45,46]. Similarly, the possibly of inhibiting two targets, possibly involved in different cellular pathways, could result in important achievements, especially in terms of drug resistance and therapeutic efficacy [47,48,49,50].

In conclusion, proteases are a valid target for the development of new effective drugs for a large panel of diseases. The great efforts of the scientific community have provided significant findings in this research field. However, the drug translation process remains a significant challenge for many reasons, such as cytotoxicity, poor drug-like properties, and a lack of novelty with respect to the available drugs. In the future, rational and aimed endeavors should be made to solve these limitations.

## Abbreviations

The following abbreviations are used in this editorial:

WHO	World Health Organization
ACE	Angiotensin-converting enzyme
CPDO	Chronic Obstructive Pulmonary Disease
DPP-4	Dipeptidyl peptidase 4
SASR-CoV-2	Severe Acute Respiratory Syndrome COronaVirus 2
COVID-19	COronaVIrus Disease 19
HIV	Human Immunodeficiency Virus
